# Continuation of subcutaneous or intramuscular injectable contraception when administered by facility-based and community health workers: findings from a prospective cohort study in Burkina Faso and Uganda^☆^

**DOI:** 10.1016/j.contraception.2018.08.007

**Published:** 2018-11

**Authors:** Ellen MacLachlan, Lynn M Atuyambe, Tieba Millogo, Georges Guiella, Seydou Yaro, Simon Kasasa, Justine Bukenya, Agnes Nyabigambo, Fredrick Mubiru, Justine Tumusiime, Yentéma Onadja, Lonkila Moussa Zan, Clarisse Goeum/Sanon, Seni Kouanda, Allen Namagembe

**Affiliations:** a PATH, PO Box 900922, Seattle, WA 98109, USA; b Makerere University, College of Health Sciences, School of Public Health, PO Box 7072, Kampala, Uganda; c Institut Africain de Santé Publique (IASP), 12 BP 199, Ouagadougou; d Institut Supérieur des Sciences de la Population (ISSP), Université Ouaga I Pr Joseph Ki-Zerbo, 03 BP 7118, Ouagadougou 03, Burkina Faso; e Centre MURAZ, 01 BP 390, Bobo-Dioulasso 01, Burkina Faso; f FHI 360, Plot 15 Kitante Close, Kampala, Uganda; g PATH, PO Box 7404, Kampala, Uganda; h Institut de Recherche en Sciences de la Santé (IRSS), BP 7192, Ouagadougou, Burkina Faso

**Keywords:** DMPA-SC, DMPA-IM, Injectable contraception, Community-based distribution, Sayana Press

## Abstract

**Objective:**

The aim of this study was to examine continuation of subcutaneous and intramuscular depot medroxyprogesterone acetate (DMPA-SC and DMPA-IM) when administered by facility-based health workers in Burkina Faso and Village Health Teams (VHTs) in Uganda.

**Study design:**

Participants were family planning clients of health centers (Burkina Faso) or VHTs (Uganda) who had decided to initiate injectable use. Women selected DMPA-SC or DMPA-IM and study staff followed them for up to four injections (providing 12 months of pregnancy protection) to determine contraceptive continuation. Study staff interviewed women at their first injection (baseline), second injection, fourth injection and if they discontinued either product.

**Results:**

Twelve-month continuation in Burkina Faso was 50% for DMPA-SC and 47.4% for DMPA-IM (p=.41, *N*=990, 492 DMPA-SC and 498 DMPA-IM). Twelve-month continuation in Uganda was 77.8% for DMPA-SC and 77.4% for DMPA-IM (p=.85, *N*=1224, 609 DMPA-SC and 615 DMPA-IM). Reasons for discontinuation of DMPA across groups in Burkina Faso included side effects (90/492, 18.3%), being late for injection (68/492, 13.8%) and refusal of spouse (51/492, 10.4%). Reasons for discontinuation in Uganda included being late for injection (65/229, 28.4%), received from non-VHT (50/229, 21.8%) and side effects (34/229, 14.8%). Increased age (adjusted hazard ratio=0.98, p=.01) and partner acceptance of family planning (adjusted hazard ratio=0.48, p<.001) had protective effects against discontinuation in Burkina Faso; we did not find statistically significant variables in Uganda.

**Conclusions:**

There is no difference in 12-month continuation (through four injections) between DMPA-SC and DMPA-IM whether from facility-based health workers in Burkina Faso or VHTs in Uganda. Continuation was higher through community-based distribution in Uganda than health facilities in Burkina Faso.

**Implications:**

The subcutaneous formulation of depot medroxyprogesterone acetate (DMPA-SC) is increasingly available in Family Planning 2020 countries. Use of DMPA-SC does not appear to change continuation relative to traditional intramuscular DMPA. Growing evidence of DMPA-SC's suitability for community-based distribution and self-injection may yield indirect benefits for contraceptive continuation and help reach new users.

## 1 Introduction

Injectable contraceptives are among the world's most widely used methods for preventing pregnancy, offering women safe and effective protection, convenience and privacy [1]. Depot medroxyprogesterone acetate (DMPA) is the most commonly used injectable contraceptive; health care providers customarily administer the drug intramuscularly (DMPA-IM; brand name: Depo-Provera®, by Pfizer Inc.). However, a new, lower-dose, subcutaneous version of DMPA (DMPA-SC) is increasingly available [2]. Countries in Europe and several Family Planning 2020 (FP2020) countries are currently introducing DMPA-SC. Sayana® Press, packaged in the BD Uniject™ injection system which combines the needle and drug reservoir in a single device, is the branded DMPA-SC product available to FP2020 countries. Because of the simplified injection system, a wide range of health workers — including community health workers (CHWs) — can readily administer Sayana Press to clients, and women can administer it themselves through self-injection [3], [4], [5], [6].

Burkina Faso and Uganda are among several countries that have recently introduced DMPA-SC. The contraceptive prevalence rate in Burkina Faso is low (around 15% of women of reproductive age are using contraceptive methods) and has been slow to increase [7]. Of the women using modern contraception, 32% use injectables [8]. The total fertility rate remains high with important disparities between rural and urban areas (respectively, 6.7 children per woman vs. 3.9 children per woman), and the maternal mortality rate is among the highest in the world (341 per 100,000 alive newborns) [7]. In order to improve reproductive health outcomes in the country, the government set the status of women as a priority intervention in the national health policy and, in 2013, adopted a plan for continued repositioning of family planning. Sayana Press received regulatory approval in 2013, and health facilities introduced it across four regions [9]. In 2016, the introduction moved to national scale-up.

Contraceptive use is higher in Uganda (30% prevalence), with injectables being the most commonly used method among married and unmarried women [10]. In response to WHO task sharing guidance, the Ministry of Health authorized CHWs called Village Health Teams (VHTs) to provide family planning products, including DMPA injections, to women in their communities in 2013. Following this agreement and national regulatory approval of Sayana Press, and building on the Ugandan government's goal to expand contraceptive access into rural communities, the Ministry of Health introduced VHT-delivered DMPA-SC in 28 districts in September 2014. Programmatic results (Fredrick Mubiru, written communication, April 2018) have shown that VHTs can effectively administer the injectables with minimal challenges and that uptake of injectables was 11% higher in areas where community-based distribution occurred compared to areas where only facility-based administration was available. Currently, national scale-up of DMPA-SC is under way. Program monitoring data from Uganda's introduction found that health workers administered 29% of DMPA-SC doses to new users of family planning, thereby helping to address unmet need [11].

Addressing unmet need for family planning requires supporting women to continue using contraception for as long as they wish to do so, in addition to reaching new users of contraception [12]. Method-related concerns, primarily side effects or myths and rumors, are a predominant reason for discontinuation, especially among those with unmet need for contraception [13]. The two studies described in this article intended to explore whether DMPA-SC could help to address unmet need by reducing discontinuation of injectable contraception relative to DMPA-IM. PATH and partners conducted two contraceptive continuation studies from December 2015 through April 2017 to determine DMPA-SC and DMPA-IM contraceptive continuation over four injections (approximately 12 months) when each product was delivered by facility-based health workers in Burkina Faso or VHTs in Uganda. These studies therefore present the first comparison of 12-month continuation rates for DMPA-SC and DMPA-IM when administered by health workers and, as a secondary objective, present reasons for discontinuation for each product.

## 2 Methods

### 2.1 Study population and design

In Burkina Faso, we conducted the study in four regions (Centre, Centre-Ouest, Boucle du Mouhoun and Hauts-Bassins). Study participants were newly initiating injectable contraceptive users from 10 participating health facilities in each of the 4 regions. Of 40 participating facilities, 31 were Centres de Santé et de Promotion Sociale, the most peripheral level of medical care available, serving the most remote areas of Burkina Faso and covering a population of 8000 to 15,000. Seven facilities in the study were nongovernmental organization (NGO) family planning clinics administered by either Marie Stopes Burkina Faso or L'Association Burkinabè pour le Bien-Être Familial and located in the larger cities of Ouagadougou, Bobo Dioulasso and Koudougou. The remaining two study sites were larger medical centers.

In Uganda, we implemented the study through VHTs (and their associated government health center or NGO) in six districts (Busia, Mubende, Gulu, Mayuge, Kayunga and Oyam). A total of 27 health facilities participated (one NGO facility; the remaining, government facilities), with the affiliated VHTs recruiting community-based clients who were newly initiating injectable contraception into either the DMPA-SC or DMPA-IM group.

In both study settings, we carried out a prospective cohort study. Women seeking injectable contraception at a participating site self-selected DMPA-SC or DMPA-IM. All health workers involved in the study counseled women on both injectables and provided DMPA-SC or DMPA-IM based on the client's preference; study staff provided additional training and supervision to minimize the potential for provider bias. After giving the injectable contraceptive to the woman, the health worker asked her interest in participating in the study using a recruitment script and eligibility checklist. A woman was eligible if she was 18 to 49 years old (15 to 49 in Uganda), had no contraindications to DMPA, was newly initiating the injectable at the time of enrollment (either a new user of injectables or a woman who had used injectables in the past but had stopped), planned to reside in the area for the 12 months, expressed a desire to prevent pregnancy for at least 12 months and provided informed consent. Study staff noted the injection schedule for each woman so that research assistants could plan to return to interview the women at specific time points. Research assistants then followed up with eligible women who had indicated a willingness to participate, explained the study further, and enrolled them as a DMPA-SC or DMPA-IM user in the study if they agreed to participate. Enrollment in both studies began in December 2015 and continued through April 2016.

Research assistants followed participants for up to four injections (providing approximately 12 months of pregnancy protection), concluding in April 2017. We describe more information on follow-up procedures in Section 2.4.

### 2.2 Ethics approvals

The Ministry of Health Comité d'Ethique pour la Recherche en Santé approved the Burkina Faso study on October 7, 2015. The Higher Degrees, Research and Ethics Committee of Makerere University College of Health Sciences School of Public Health and the Uganda National Council for Science and Technology approved the Uganda study on November 2, 2015.

### 2.3 Sample size

We calculated sample sizes with the intent to observe whether or not there was a significant difference in 12-month injectable continuation use between DMPA-IM and DMPA-SC users. The target sample size for the Burkina Faso study (483 women per group) assumes a statistical power of 90%, a significance level of .05 and a 10-percentage-point difference in continuation rates between DMPA-IM users (an estimated 72% [7]) and those receiving DMPA-SC from a facility-based health worker. We used a 20% loss to follow-up rate.

The target sample size for the Uganda study (604 women per group) assumes a statistical power of 87%, a significance level of .05 and a 10-percentage-point difference in continuation rates between DMPA-IM users (54% continuation in Uganda, based on 2011 Demographic and Health Survey data [14]) and those receiving DMPA-SC from a VHT. We used a 20% loss to follow-up rate for Uganda as well, a typical rate for community-based studies.

### 2.4 Data collection

Data collection methods were equivalent across countries and sites. Following informed consent and their first DMPA injection, research assistants interviewed women in as quiet and private a location as possible. A trained research assistant fluent in the local language conducted a baseline interview using a questionnaire translated into the local language for that district or region. The baseline interview collected background information and assessed women's reasons for seeking an injectable. Research assistants collected study data on tablets that used a mobile application of Research Electronic Data Capture tools. After the interview, research assistants asked women for detailed contact information for follow-up and noted the next injection appointment date for each enrolled woman. The study compensated women for their transport costs.

Study staff followed women for up to four injections (providing 12 months of pregnancy protection) and performed detailed follow-up surveys at the second and fourth injections or upon discontinuation. Health providers kept track of whether women enrolled in the study returned for their second, third and fourth injections on time, and alerted research assistants about which participants returned for their reinjections within the 30-day window and which did not. Research assistants then proceeded to contact participants, either by phone or in person as preferred by the participants at baseline, to conduct the follow-up or discontinuation surveys (this only occurred after the 30-day window for reinjection had closed).

Follow-up interviews documented women's second and fourth injections and asked women about their experience and satisfaction with either method. At 6 months, if women received their third injection, research assistants noted this, although they did not conduct interviews in order to preserve women's time. Women who stopped the injectable or who received injections after the close of a 30-day injection window were asked to respond to a discontinuation survey. Discontinuation surveys asked participants about their reasons for discontinuation, including their personal satisfaction with the chosen method, any changes to their lifestyle that occurred and practical issues that are common barriers to continuation (e.g., costs of transport, time to reach the health worker). Special 3- and 9-month surveys were created for women who switched from one DMPA injectable to the other.

### 2.5 Statistical analysis

First, we assessed baseline characteristics of study participants for comparability between DMPA-SC and DMPA-IM groups using *t* test for continuous covariates and *χ*^2^ tests for categorical covariates. Second, we calculated continuation rates of DMPA-SC and DMPA-IM as the proportion of women who completed four consecutive injections providing 12 months of pregnancy protection. Women who did not receive their second, third or fourth injection were considered discontinuers. We calculated continuation rates based on the principle of intention to treat. For the analysis, we classified women lost to follow-up as discontinuers in their original group and women who switched injectable types during the study as continuers in their original injectable group. We also assessed the probability of continuation over four injections using a Kaplan–Meir estimator, also known as a survival analysis. We used the time to discontinuation as the outcome variable for the survival analysis. Finally, we ran Cox proportional-hazards regression models to determine the factors associated with the risk of discontinuation of DMPA. We chose factors to include in the final Cox hazard models based on significance in crude hazard ratios for discontinuation and theoretical associations with discontinuation of injectable contraceptives in these settings.

## 3 Results

### 3.1 Description of participants

In Burkina Faso, 492 women enrolled in the DMPA-SC group and 498 in the DMPA-IM group. There were several statistically significant differences between groups; women who chose DMPA-SC were slightly younger, had lower parity and were more likely to currently be in school. In addition, women who chose DMPA-SC were less likely to be new users of family planning and were more likely to have previously used injectable contraception. Lastly, DMPA-SC users reported a higher number of household assets (Table 1).

**Table 1:** Demographic characteristics and family planning experience of women who chose to use DMPA-SC and DMPA-IM in Burkina Faso and Uganda

Burkina Faso	DMPA-SC (*N*=492)	DMPA-IM (*N*=498)	p value
Mean age (SD)	26.8 (6.7)	28.6 (7.4)	.00
Mean parity (SD)	2.9 (1.8)	3.2 (1.9)	.02
Marital status, *n* (%)			
Married/in union	404 (82.1)	427 (85.7)	.12
Not in union	88 (17.9)	71 (14.3)	
Currently in school, *n* (%)	69 (14.0)	44(8.8)	.03
Mean number of household assets (SD)	8.3 (4.1)	7.7 (3.9)	.04
New users of family planning, *n* (%)	242 (49.2)	322 (64.7)	.00
Previously used injectables, *n* (%)	121 (24.6)	89 (17.9)	.01
Injection anxiety, *n* (%)			
Very anxious	73 (14.8)	47 (9.4)	.00
A little anxious	110 (22.4)	81 (16.3)	
Not at all anxious	309 (62.8)	370 (74.3)	
Husband supports use of family planning, *n* (%)	377 (76.6)	371 (74.5)	.44
Family planning decisions made jointly, *n* (%)	105 (21.3)	116 (23.3)	.43


Uganda	DMPA-SC (*N*=609)	DMPA-IM (*N*=615)	p value
Mean age (SD)	25.8 (6.3)	26.4 (6.6)	.10
Mean parity (SD)	1.7 (2.1)	1.6 (2.1)	.80
Marital status, *n* (%)			
Married/in union	501 (82.3)	514 (83.6)	.54
Not in union	108 (17.7)	101 (16.4)	
Currently in school, *n* (%)	22 (3.6)	16 (2.6)	.49
Mean number of household assets (SD)	8.6 (4.3)	8.4 (4.5)	.47
New users of family planning, *n* (%)	383 (62.9)	400 (65.0)	.43
Previously used injectables, *n* (%)	160 (26.3)	157 (25.5)	.60
Injection anxiety, *n* (%)			
Very anxious	79 (13.0)	69 (11.2)	.04
A little anxious	98 (16.1)	70 (11.4)	
Not at all anxious	331 (54.4)	376 (61.1)	
Husband supports use of family planning, *n* (%)	395 (64.9)	404 (65.7)	.76
Family planning decisions made jointly, *n* (%)	185 (30.4)	155 (25.2)	.02

Baseline characteristics were similar between DMPA-IM and DMPA-SC groups in Uganda, although DMPA-SC users were more likely to report that they made family planning decisions jointly (Table 1). Across both countries, DMPA-SC users were more likely to report being very anxious or a little anxious about injections compared to DMPA-IM users (Table 1).

**Fig. 1:**
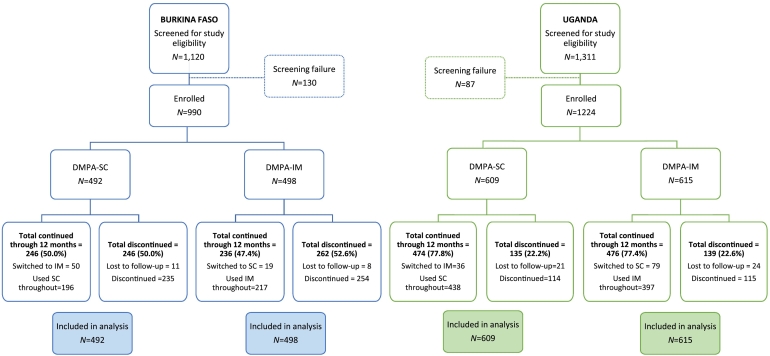
Participant flow diagram for DMPA-SC and DMPA-IM users enrolled in the study, and injectable continuation rates in Burkina Faso and Uganda.

We describe the number of women contacted, enrolled in each group and included in the analysis in Fig. 1.

### 3.2 Comparison of continuation rates of DMPA-IM and DMPA-SC

**Fig. 2:**
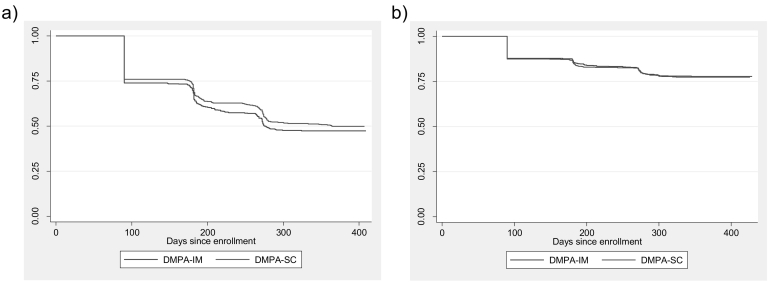
Kaplan–Meier cumulative probability of DMPA-SC and DMPA-IM continuation in (A) Burkina Faso and (B) Uganda. Log-rank test for equality of survivor function, p value=.31 (Burkina Faso) and .88 (Uganda).

Over the study period in Burkina Faso, the study lost a total of 19 women to follow-up (1.9%, *n*=990): 11 in the DMPA-SC group and 8 in the DMPA-IM group. In all, 50 women switched from DMPA-SC to DMPA-IM, and 19 women switched from DMPA-IM to DMPA-SC (two women switched at both the second and fourth injections). Overall, 50.0% of the DMPA-SC group continued for four injections (approximately 12 months) compared to 47.4% of the DMPA-IM group (p=.41; Fig. 1). The probability of continuation was slightly higher for DMPA-SC at the second, third and fourth injections, although it was not statistically significant (Fig. 2A).

In Uganda, the study lost a total of 45 women to follow-up (3.7%, *n*=1224): 21 in the DMPA-SC group and 24 in the DMPA-IM group. A total of 36 women switched from DMPA-SC to DMPA-IM, and 79 women switched from DMPA-IM to DMPA-SC (9 women switched at both the second and fourth injections). Overall, 77.8% of the DMPA-SC group continued through four injections compared to 77.4% of the DMPA-IM group (p=.85; Fig. 1). The probability of continuation was almost identical for DMPA-SC and DMPA-IM in Uganda at all time points (Fig. 2B).

As described in the methods, we categorized women who switched injectable types as continuers in their original injectable groups for the analysis. In both countries, when we reassigned women who switched (for any reason other than stockout) to the group to which they switched to, the continuation rates remained similar, and the difference was nonsignificant.

### 3.3 Factors associated with discontinuation

In Burkina Faso, when considering multiple factors that could be linked to discontinuation, under the hazard ratio model, age was a moderate risk factor for discontinuation; as age increased, the risk of discontinuation decreased by 2% (adjusted hazard ratio=0.98, 0.96–0.99, p=.01). Partner acceptance of family planning was highly predictive of discontinuation. Having a partner who approved of contraceptive use decreased the risk of discontinuation by 52% even when controlling for other factors in the multivariate hazard ratio model (adjusted hazard ratio=0.48, 0.34–0.68, p<.001) (Table 2).

**Table 2:** Crude and adjusted hazard ratios predicting factors associated with the risk of discontinuation of DMPA-SC and DMPA-IM over four injections in Burkina Faso (*N*=990) and Uganda (*N*=1224)

Burkina Faso	Crude HR (95% CI)	p > |*z*|	Adjusted HR (95% CI)	Multivariate p
Injectable group DMPA-SC	0.92 (0.72–1.2)	.50	1.0 (0.81–1.4)	.69
Age in years	0.99 (0.97–1.0)	.09	0.98 (0.96–0.99)	.01
Number of children	0.92 (0.86–0.98)	.01	0.98 (0.88–1.1)	.67
Public-sector health facility	0.75 (0.53–1.1)	.11	0.88 (0.58–1.3)	.55
Woman works outside home	1.3 (0.99–1.6)	.07	1.1 (0.83–1.4)	.57
Married	0.71 (0.52–0.97)	.03	0.99 (0.66–1.5)	.95
Fertility desire				
No more children	1 (reference)		1 (reference)	
More children	0.89 (0.61–1.3)	.52	0.65 (0.41–1.0)	.06
Undecided	1.5 (0.98–2.2)	.06	0.94 (0.58–1.4)	.78
Partner accepts family planning	0.52 (0.37–0.72)	<.001	0.48 (0.34–0.68)	<.001
Anxiety about injections	1.4 (0.95–1.9)	.09	1.1 (0.77–1.7)	.52
Paid for transport to health facility	1.3 (0.92–1.8)	.13	2.1 (0.86–5.0)	.11
Concerns about DMPA use	1.3 (0.93–1.7)	.14	1.1 (0.80–1.6)	.51
Amount paid for transport	1.0 (0.99–1.0)	.13	1.0 (1.0–1.0)	.29


Uganda	Crude HR (95% CI)	p > |*z*|	Adjusted HR (95% CI)	Multivariate p
Injectable group DMPA-SC	0.94 (0.66–1.3)	.72	0.81 (0.54–1.2)	.32
Age in years	0.98 (0.95–1.0)	.11	0.99 (0.94–1.0)	.60
Concerns about DMPA	1.7 (1.1–2.5)	.01	1.1 (0.66–1.9)	.69
Spouse/partner has other partners	1.3 (0.89–2.0)	.18	1.5 (0.94–2.3)	.09
Has used injectables in the past	1.3 (0.89–1.9)	.18	1.1 (0.69–1.9)	.62
Fertility desire				
No more children	1 (reference)		1 (reference)	
More children	1.8 (1.1–2.9)	.02	1.5 (0.72–3.0)	.30
Undecided	0.39 (0.09–1.7)	.20	1.2 (0.27–5.7)	.77
Partner accepts family planning	0.66 (0.44–0.98)	.04	0.68 (0.43–1.1)	.10
Paid for transport to health facility	1.4 (0.89–2.1)	.15	1.3 (0.81–2.0)	.30
Both injectables available	1.6 (1.1–2.4)	.02	0.81 (0.48–1.3)	.41
Side effects from injectables in past	1.6 (1.1–2.5)	.03	1.2 (0.67–2.3)	.48
Household goods score	0.96 (0.92–1.0)	.05	1.0 (0.92–1.1)	.96
Number of children	0.90 (0.82–0.99)	.03	0.87 (0.75–1.0)	.08

Abbreviations: CI, confidence interval; HR, hazard ratio.

In Uganda, no single factor predicting discontinuation remained significant when adjusted for other variables despite many crude hazard ratios that were statistically significant (Table 2).

### 3.4 Reported reasons for discontinuation

The most commonly reported principle reasons for discontinuation of DMPA in Burkina Faso were side effects (18.3%), being late for injection (13.8%) and refusal of spouse (10.4%). Reasons for discontinuation were similar across groups (Table 3). A total of 21 women reported that they received their injections at a different health facility in Burkina Faso, although as per the study design, they were considered to have discontinued. In Uganda, principle reasons for discontinuation were being late for an injection (28.4%), received injection from a non-VHT (21.8%) and side effects (14.8%). As there was no way to confirm injections administered elsewhere, we considered 50 women who reported receiving their injection from some place other than a VHT (e.g., a local clinic) as discontinuers. If women who reported receiving their injection elsewhere are included in the continuation rates, the rates by group remain similar and the difference nonsignificant. Women's primary reasons for discontinuation were similar across the DMPA-IM and DMPA-SC groups (Table 3).

**Table 3:** Women's self-reported reasons for discontinuation of DMPA-SC and DMPA-IM in Burkina Faso and Uganda

Burkina Faso	DMPA-SC (*N*=232)	DMPA-IM (*N*=260)	Total (*N*=492)
Side effects	17.7% (41)	18.9% (49)	18.3% (90)
Late for injection	15.1% (35)	12.7% (33)	13.8% (68)
Spouse opposed	10.3% (24)	10.4% (27)	10.4% (51)
Traveling/away/farming	12.1% (28)	6.9% (18)	9.4% (46)
To have a baby	8.2% (19)	10.0% (26)	9.2% (45)
Switched to other family planning method	9.9% (23)	7.7% (20)	8.7% (43)
No sexual relations	6.0% (14)	5.4% (14)	5.7% (28)
Forgot	4.3% (10)	6.5% (17)	5.5% (27)
Difficulty getting to health facility	4.7% (11)	4.2% (11)	4.5% (22)
Concerns about long-term health effects or fertility effects	2.2% (5)	4.2% (11)	3.3% (16)
Ill or family member ill	3.0% (7)	1.2% (3)	2.0% (10)
Wanted to stop hormones	0.9% (2)	1.9% (5)	1.4% (7)
Got pregnant	1.7% (4)	1.2% (3)	1.4% (7)
Other	3.9% (9)	8.9% (23)	6.5% (32)


Uganda	DMPA-SC (*N*=113)	DMPA-IM (*N*=116)	Total (*N*=229)
Late for injection	27.4% (31)	29.3% (34)	28.4% (65)
Received from non-VHT	20.4% (23)	23.3% (27)	21.8% (50)
Side effects	15.0% (17)	14.7% (17)	14.8% (34)
No sexual relations	5.3% (6)	12.9% (15)	9.2% (21)
Spouse opposed	8.8% (10)	6.0% (7)	7.4% (17)
Got pregnant	4.4% (5)	6.0% (7)	5.2% (12)
To have a baby	7.1% (8)	3.4% (4)	5.2% (12)
Switched to other family planning method	5.3% (6)	0.9% (1)	3.1% (7)
Difficulty getting to VHT/no VHT/stockout	1.8% (2)	1.7% (2)	1.8% (4)
Concerns about long-term health effects or fertility effects	1.8% (2)	0.9% (1)	1.3% (3)
Other	2.7% (3)	0.9% (1)	1.8% (4)

## 4 Discussion

These findings contribute to emerging literature on contraceptive discontinuation, specifically adding the first-ever data on women's continuation of the more recently introduced injectable contraceptive, DMPA-SC, compared to the more traditional injectable, DMPA-IM, when both products are administered by health workers. Given a new 6-year agreement announced in 2017 that guarantees near parity in price between DMPA-IM and DMPA-SC for qualified buyers (e.g., governments in FP2020 countries), there is even greater interest in understanding the potential benefits of this new product option. Overall, these injectable continuation studies in Burkina Faso and Uganda indicate that the two DMPA injectable types are likely to have very similar continuation rates when delivered by the same health workers. At the same time, due to the product's ease of use, introduction of DMPA-SC holds particular potential to catalyze investments in and use of delivery options such as community-based distribution and self-injection of contraception, and increase access for women.

This study also yields insights relevant to delivery of injectable contraception more generally and links to contraceptive continuation. According to a multicountry analysis, the median duration of injectable contraceptive use is 11.9 months — lower than for IUDs, condoms, pills and traditional methods — and the probability of continuation of injectable contraception at 12 months is 59.4% [15]. Relative to this finding, women in the Burkina Faso study who received either DMPA product from clinic-based health workers had low injectable continuation rates (about 50%). The continuation rates in the Uganda study were higher than in Burkina Faso and global averages, potentially reflecting an added benefit of injectable contraceptive administration by VHTs. Women may be more likely to continue DMPA injections if they are able to visit a VHT located near their village for reinjections. This aligns with previous findings from Kenya [16] and from early work with VHTs in Uganda [18]: that offering injectable contraception through CHWs seems to increase continuation by improving client satisfaction with the method and with contraceptive services overall. In addition, a concurrent study that explored self-injection continuation in Uganda found lower continuation rates for facility-based health worker administration of DMPA-IM than what was reported here for delivery of both DMPA products by VHTs [17]. Training, equipping and supporting CHWs to provide a wide range of family planning methods are proven, high-impact practices in family planning [18]. While interventions to improve quality of services and train VHTs were ongoing at the time of this study and could also account for the relatively high continuation rates observed, Burkina Faso made new investments in clinic-based health worker training in its intervention areas as well.

Information from these studies on women's reported reasons for discontinuation suggests that we can do more to support women who wish to continue using contraception for longer periods of time. Similar to previous findings that many women discontinue injectables for method-related reasons (such as side effects or myths) [13], we found that the experience of side effects was a major reason for discontinuation in both countries. These results reinforce the importance of offering a wide range of contraceptive options to enable switching and providing adequate counseling and education about side effects. In Burkina Faso, late injections due to difficulty getting to a health facility due to travel, family matters, work or farming were common and contributed to the low continuation rates — and even with the high continuation rates in Uganda, women were sometimes more than 30 days late for their injections. Health care workers could work with women during contraception counseling sessions to formulate a plan for receiving on-time injections, and mobile health reminder systems could be explored for injections in places with consistent phone coverage [18].

Studies have demonstrated that the efficacy, safety and immediacy of contraceptive effect of DMPA-SC are equivalent to DMPA-IM; tolerability of the product is generally expected to be comparable as well. Perhaps, then, the lack of continuation differences when the same health workers deliver the two products is not surprising. At the same time, previous research shows that injectable contraception users and health workers, including CHWs, in Uganda and Senegal preferred DMPA-SC over DMPA-IM [4], [5]. New research from Malawi, Uganda and the United States has shown that women who self-inject DMPA-SC have significantly higher continuation than those who receive DMPA injections from health workers [19], [20], [21]. Taken all together, these results indicate that at least some challenges with injectable contraceptive continuation may be surmountable by making delivery more convenient and accessible for women.

### 4.1 Study limitations

We excluded women already using injectable contraception from participation in order to calculate continuation from the first injection for all women; therefore, our samples may not be representative of injectable contraceptive users in these countries. Additionally, we designed these studies to compare continuation between two DMPA injectable products; some discontinuers switched to other contraceptive methods, so DMPA discontinuation may not translate to changes in broader measures like contraceptive prevalence rates and unmet need for family planning.

## References

[1] United Nations, Department of Economic and Social Affairs, Population Division (2013). Trends in contraceptive methods use worldwide.

[2] PATH (2018). DMPA-SC: expanding contraceptive access and options. https://www.path.org/articles/dmpa-sc/.

[3] Dragoman M.V., Gaffield M.E. (2016). The safety of subcutaneously administered depot medroxyprogesterone acetate (104mg/0.65mL): a systematic review. Contraception.

[4] Burke H.M., Mueller M.P., Packer C., Perry B., Bufumbo L., Mbengue D. (2014). Provider acceptability of Sayana® Press: results from community health workers and clinic-based providers in Uganda and Senegal. Contraception.

[5] Burke H.M., Mueller M.P., Perry B., Packer C., Bufumbo L., Mbengue D. (2014). Observational study of the acceptability of Sayana® Press among intramuscular DMPA users in Uganda and Senegal. Contraception.

[6] Pfizer Inc (2015). Pfizer's Sayana® Press becomes first injectable contraceptive in the United Kingdom available for administration by self-injection [press release]. https://www.pfizer.com/news/press-release/press-release-detail/pfizer_s_sayana_press_becomes_first_injectable_contraceptive_in_the_united_kingdom_available_for_administration_by_self_injection.

[7] Institut National de la Statistique et de la Démographie (INSD) Burkina Faso, ICF International (2012). Enquête Démographique et de Santé et à indicateurs multiples du Burkina Faso 2010. https://dhsprogram.com/pubs/pdf/FR256/FR256.pdf.

[8] Ministère de la santé (2017). Plan National d'Accélération de Planification Familiale du Burkina Faso 2017–2020. http://ec2-54-210-230-186.compute-1.amazonaws.com/wp-content/uploads/2017/11/Burkina-Faso-CIP-2017.pdf.

[9] Guiella G., Shani T., Hamadou C., Radloff S., Yoonjoung C. (2018). Rapid uptake of the subcutaneous injectable in Burkina Faso: evidence from PMA2020 cross-sectional surveys. Glob Health Sci Pract.

[10] Uganda Bureau of Statistics (UBOS), ICF (2018). Uganda demographic and health survey 2016.

[11] Stout A., Wood S., Barigye G., Kabore A., Siddo D., Ndione-Colli I. (2018). Expanding access to injectable contraception: results from pilot introduction of subcutaneous DMPA (DMPA-SC) in four African countries. Glob Health Sci Pract.

[12] Jain A.K. (1989). Fertility reduction and the quality of family planning services. Stud Fam Plann.

[13] Castle S., Askew I. (2015). Contraceptive discontinuation: reasons, challenges and solutions. Family Planning 2020 and Population Council. http://www.familyplanning2020.org/microsite/contraceptive-discontinuation.

[14] Uganda Bureau of Statistics (UBOS), ICF International (2012). Uganda demographic and health survey 2011. https://dhsprogram.com/pubs/pdf/fr264/fr264.pdf.

[15] Ali M.M., Cleland J.G., Shah I.H. (2012). Causes and consequences of contraceptive discontinuation: Evidence from 60 demographic and health surveys.

[16] Olawo A.A., Bashir I., Solomon M., Stanback J., Ndugga B.M., Malonza I. (2013). "a cup of tea with our CBD agent … ": community provision of injectable contraceptives in Kenya is safe and feasible. Glob Health Sci Pract.

[17] Stanback J., Mbonye A.K., Bekiita M. (2007). Contraceptive injections by community health workers in Uganda: a nonrandomized community trial. Bull World Health Organ.

[18] Labrique A.B., Vasudevan L., Kochi E., Fabricant R., Mehl G. (2013). mHealth innovations as health system strengthening tools: 12 common applications and a visual framework. Glob Health Sci Pract.

[19] Burke H.M., Chen M., Buluzi M., Fuchs R., Wevill S., Venkatasubramanian L. (2018). Effect of self-administration versus provider-administered injection of subcutaneous depot medroxyprogesterone acetate on continuation rates in Malawi: a randomized controlled trial. Lancet Glob Health.

[20] Cover J., Namagembe A., Tumusiime J., Nsangi D., Lim J., Nakiganda-Busiku D. (2018). Continuation of injectable contraception when self-injected vs. administered by a facility-based health worker: a non-randomized, prospective cohort study in Uganda. Contraception.

[21] Kohn J.E., Simons H.R., Della Badia L., Draper E., Morfesis J., Talmont E. (2018). Increased 1-year continuation of DMPA among women randomized to self-administration: results from a randomized controlled trial at Planned Parenthood. Contraception.

